# Lost circulation intensity characterization in drilling operations: Leveraging machine learning and well log data

**DOI:** 10.1016/j.heliyon.2024.e41059

**Published:** 2024-12-09

**Authors:** Ahmad Azadivash

**Affiliations:** Department of Petroleum Engineering, Amirkabir University of Technology, Tehran, Iran

**Keywords:** Lost circulation, Machine learning, Well log, Drilling operation, Kopeh Dagh

## Abstract

Lost circulation is one of the important challenges in drilling operations and bears financial losses and operational risks. The prime causes of lost circulation are related to several geological parameters, especially in problem-prone formations. Herein, the approach of applying machine learning models to forecast the intensity of lost circulation using well-log data is presented in this work. It concerns a gas field in northern Iran and contains nine well logs with lost circulation incidents categorized into six intensity classes. After rigorous exploratory analysis and preprocessing of the data, seven machine learning methods are applied: Random Forest, Extra Trees, Decision Tree, XGBoost, k-Nearest Neighbors, Support Vector Machine, and Hard Voting. Random Forest, Extra Trees, and Hard Voting are the best-performing methods. These models attained the most robust results on both key performance metrics and, hence, can predict the intensity of lost circulation quite well. Models of Extra Trees and Hard Voting show very high predictive performance values. On the other hand, their limitations in some intensity classes suggest further refinement. In this regard, the ensemble methods are highly effective for managing the multivariate nature of the task. Hard Voting aggregates multiple classifiers, becoming superior to individual models like support vector machines. This paper offers new insight into integrating machine learning to well-log data toward enhancing lost circulation prediction by offering a dependable foundation for real-time drilling decision-making. These results show that the models have the potential to lower operational risks, improve drilling safety, and minimize nonproductive time; hence, they form a quantum leap in lost circulation control.

## 1 Introduction

Lost circulation is a difficult problem with broad consequences that presents a major challenge to the discipline of drilling engineering. The oil and gas sector's constant search for deep and ultra-deep reservoirs has recently drawn more attention to lost circulation [1,2]. This problem affects drilling activities significantly; it causes prolonged periods of non-production, delays in construction schedules, and increased risk connected to well control [[3], [4], [5], [6], [7]]. Also, lost circulation causes drilling fluids and plugging materials to be used up too quickly, which costs a lot of money and causes a chain of complex problems like well collapses, sticking, and blowouts [2,8]. The underlying reasons for lost circulation often trace their origins to geological formations characterized by a limited range of suitable mud weights. This happens a lot in different geological places, like reservoirs where the pressure has dropped, broken areas, long horizontal well sections, and shallow formations in very deep water [9]. Prior scholars have extensively examined and expounded upon these contributing factors [[9], [10], [11]]. They encompass a diverse range of factors, including but not limited to the wellbore and drill string's geometric characteristics, the rheological attributes of drilling mud, the sealing capabilities of drilling mud, the effectiveness of debris removal within the wellbore, the prevailing formation pressure, fracture threshold, reservoir permeability, borehole stress distributions, the presence of open fractures and voids, elevated drilling-fluid pump pressures, substantial drilling-fluid flow rates, and the properties of the drilling fluid itself [9,12,13].

The properties of drilling fluid play a major role in the severity and possibility of lost circulation. The most influential property of mud is its density. Mud density directly relates to wellbore pressure, whether that pressure exceeds the fracture gradient of the surrounding formation. A mud density that is too high will lead to the fracturing of the formation with large volumes of mud losses. At the same time, a value that is too low will make the wellbore unstable, causing the possibility of fluid influx. It is, therefore, fundamental to lost circulation mitigation that the appropriate mud density be maintained within an operational mud window [[9], [10], [11]]. Further, viscosity affects the fluid's ability to transport cuttings, suspend solids, and seal fractures. Low-viscosity mud may not be able to plug fractures, which leads to larger mud losses effectively. In contrast, very high-viscosity mud can hinder circulation and increase the risk of lost circulation in fragile formations. Filtration losses are other important parameters of lost circulation, which considerably influence the lost circulation in porous and fractured formations. A high rate of filtration loss indicates that the mud fluid easily penetrates the formation, leaving the solids behind to promote fluid loss further. Such filtration loss, duly controlled with the use of appropriate additives, allows filter cake formation on wellbore walls and minimizes the rate of invasion into the formation to reduce the risk of mud losses. Drilling fluid properties like these must be closely monitored and adjusted according to real-time conditions met downhole since their interaction with geological formations may be critical for the volume and intensity of the lost circulation events. This will give a chance to avoid better or considerably reduce severe losses in circulation and improve the stability of the wellbore by precise control of mud density, viscosity, and filtration characteristics [[10], [11], [12]].

**Fig. 1:**
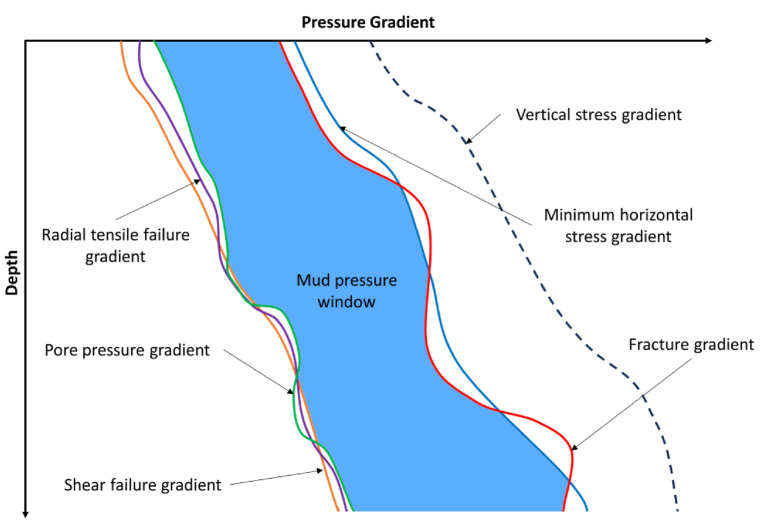
Mud window pressure gradients defining the optimal mud weight to prevent kicks and lost circulation during drilling (Adapted from Ref. [14]).

Fig. 1 illustrates the critical pressure gradients that delineate the mud weight window and help prevent lost circulation—exceeding the fracture gradient risks inducing fractures in the formation, leading to complete losses of drilling fluid. Before such losses occur, the fracture gradient determines the maximum allowable mud weight. In contrast, formation fluids can flood the wellbore and cause kicks if the mud weight is less than the pore pressure gradient. The pore pressure gradient defines the minimum mud weight required to prevent kicks. Operating between these two limiting gradients defines the muck window in which mud density can prevent kicked loss of circulation. Avoiding lost circulation, a major factor causing drilling inefficiency and cost overruns, depends on accurately defining this window for the particular formation explored [14].

Machine learning techniques have grown more and more popular recently, and they are being employed in more scientific domains, such as biology, engineering, medical research, and financial analysis. These methods have proven effective in simulating complex physical phenomena and tackling pattern recognition problems. Intelligent systems are now essential instruments in the drilling industry for solving extremely complex and nonlinear problems [[15], [16], [17], [18], [19], [20], [21], [22]].

Intelligent systems and machine learning techniques have developed as viable tools for modeling the complex physical mechanisms underpinning decreased circulation in response to this difficulty. In order to solve a variety of challenging drilling engineering challenges, machine learning has gained popularity [6,12].

Predicting lost circulation events and rates during drilling operations has proven to be a promising use of machine learning techniques, namely convolutional neural networks (CNN), random forests, support vector machines (SVM), and artificial neural networks (ANN). These models successfully predict the frequency and severity of lost circulation episodes by utilizing a variety of input variables, including as drilling parameters, geological information, mud qualities, and operational data. Prediction accuracy and real-time capabilities have continuously increased as these techniques have evolved from early ANN models to more modern implementations of sophisticated algorithms like CNN and hybrid approaches. Table 1 provides a summary of the literature on this crucial subject, demonstrating the industry's acknowledgment of machine learning's ability to reduce the danger of lost circulation and improve drilling efficiency.

**Table 1:** Comprehensive overview of published studies on lost circulation prediction to date.

Authors	Algorithms	Inputs
Moazzeni et al. [23]	ANN	Drilling data
Toreifi et al. [24]	ANN and PSO	Well coordinates, depths, geological data, mud properties
Jahanbakhshi et al. [25]	ANN	Geomechanical and operational data
Jahanbakhshi and Keshavarzi [26]	SVM	Drilling and geomechanical data
Behnoud far and Hosseini [27]	ANN and Genetic Algorithms	Drilling data
Al-Hameedi et al. [5]	Multi-regression analysis	Drilling data
Sabah et al. [28]	MLP, MLP-GA, MLP-PSO, MLP-COA, LSSVM, LSSVM-GA, LSSVM-PSO, LSSVM-COA	Drilling data
Abbas et al. [6]	ANN and SVM	Drilling data
Geng et al. [10]	Logistic Regression, Random Forest, SVM	Seismic data
Ahmed et al. [29]	ANN, Fuzzy Logic, Functional Network	Drilling data
Agin et al. [30]	Adaptive Neuro-Fuzzy Inference Systems	Drilling data
Hou et al. [11]	ANN	Drilling and lithology data
Alkinani et al. [7]	ANN	Drilling and mud logging data
Sabah et al. [31]	DTR, MLP, MLP-GA, RBF, ANFIS	Drilling data
Aljubran et al. [32]	Convolutional Neural Network (CNN)	Drilling, surface and rheology data
Wood et al. [33]	RF, ADA, DTR, CNN, LSTM, GRU, NBC, QDA, MLP, SVM	Drilling data
Pang et al. [12]	Mixture Density Networks	Mud logging data
Alsaihati et al. [34]	SVM, Random Forest, K-NN	Drilling data
Jafarizadeh et al. [35]	CNN	Drilling data

Various machine learning techniques, like ANNs, CNNs, etc., have been successfully applied based on the existing literature. However, they have yet to be leveraged well logs to predict lost circulation intensity. In this study, this gap is addressed by focusing precisely on that. The severity of lost circulation in drilling operations can be anticipated by studying well logs using machine learning algorithms. These models predict the rate at which mud is lost and enable drillers to optimize the design of drilling fluid, determine the appropriate depth for casing, identify areas with a high risk of mud loss, estimate the volume of mud needed, evaluate treatments for lost circulation, and reduce operational risk when drilling through challenging carbonate formations that are prone to significant mud losses. This approach offers a more data-driven and proactive solution, enabling precise lost circulation material selection tailored to specific well conditions. Not only is there a critical void in the literature filled by our work, but the value of data-driven lost circulation predictions in enhancing drilling efficiency, reducing non-productive time, and minimizing risk in the oil and gas industry is also underscored.

**Fig. 2:**
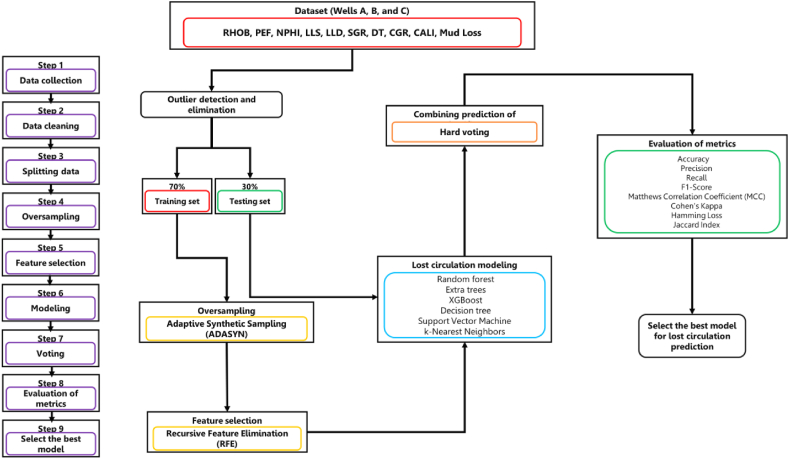
Workflow of the present research study.

Advanced machine learning algorithms, including Random Forest, Extra Trees, XGBoost, Decision Tree, Support Vector Machine (SVM), k-Nearest Neighbors (k-NN), and Hard Voting, are utilized to accomplish this task. Critical well logs such as Caliper, Computed Gamma Ray, Spectral Gamma Ray, Sonic Transit Time, Deep Laterolog Resistivity, Shallow Laterolog Resistivity, Neutron Porosity, Photoelectric Absorption Factor, and Bulk Density are utilized by these models. Lost circulation intensity is categorized into six distinct classes: No Loss (0 barrels/hour), Seepage Loss (5–10 barrels/hour), Slight Loss (10–20 barrels/hour), Moderate Loss (20–50 barrels/hour), Severe Loss (50–100 barrels/hour), and Complete Loss (Over 100 barrels/hour) [36]. Such granular classification enhances the capacity to make proactive and optimized decisions for loss mitigation during active drilling operations. The methodology for predicting lost circulation is depicted in Fig. 2.

This groundbreaking study has extensive ramifications for the oil and gas sector as it provides practical knowledge to improve effectiveness and reduce potential hazards. This work is the first to use machine learning to forecast the degree of lost circulation based on well log data. This tool is crucial for petroleum engineers and geoscientists as it greatly improves the efficiency of drilling operations. The results of this study have the potential to greatly enhance drilling efficiency and save costs by allowing for the implementation of focused and proactive measures to prevent losses in certain challenging areas. Operating in complex formations that are highly vulnerable to catastrophic mud losses has a big impact on drilling and overall operational performance. This study fills a major gap in the body of knowledge on data-driven lost circulation prediction. Through accurate and optimal management of lost circulation, it can significantly enhance drilling performance.

## 2 Geological setting

**Fig. 3:**
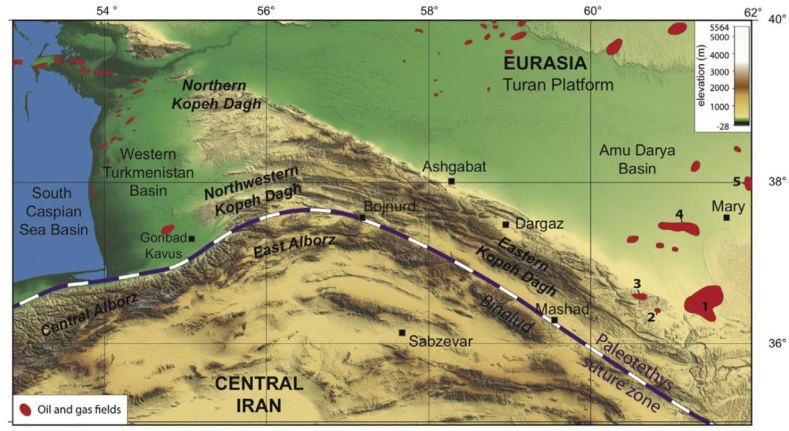
Topographic map of the Kopeh Dagh range showing locations of 6 major gas fields (1: Dauletabad; 2: Gonbadli; 3: Khangiran; 4: Shaltyk; 5: Bayram-Ali; 6: Achak) in red near the Paleotethys suture zone. Gas field data compiled from various sources [38].

The Kopeh Dagh Basin in northeastern Iran constitutes a major sedimentary basin, functioning as the southeastward continuation of the larger Amu Darya Basin spanning Turkmenistan and Uzbekistan [37,38]. Extending over 300 km from the Turkmenistan border to the Mashhad vicinity [38,39], the basin is bounded to the north by the Kopeh Dagh mountain range, formed by the convergence of the Eurasian and Iranian tectonic plates [39,40]. As the southeast fringe of the greater Amu Darya Basin, the Kopeh Dagh Basin shares a typical basement of deformed Paleozoic Hercynian rocks [39]. Significant gas reserves are hosted within Upper Jurassic carbonates and Lower Cretaceous sandstones, especially in the Khangiran gas field between Mashhad and Turkmenistan [38]. The topography of the Kopeh Dagh range and significant gas field locations are depicted in Fig. 3.

In the Late Cretaceous period, the sedimentary units were uplifted and folded as a result of the northward movement of the Iranian plate and the closing of the Neotethys Ocean [41,42]. Further compaction and consolidation took place during the Paleocene and Eocene periods, leading to the formation of shallow marine carbonate and marl deposits. The primary subsidence occurred during the Oligocene and Miocene when the Iranian and Eurasian plates clashed, resulting in flexural sinking and the deposition of thick terrestrial layers. Total sediment thickness exceeds 12 km in the basin's depocenter [38,42,43].

**Fig. 4:**
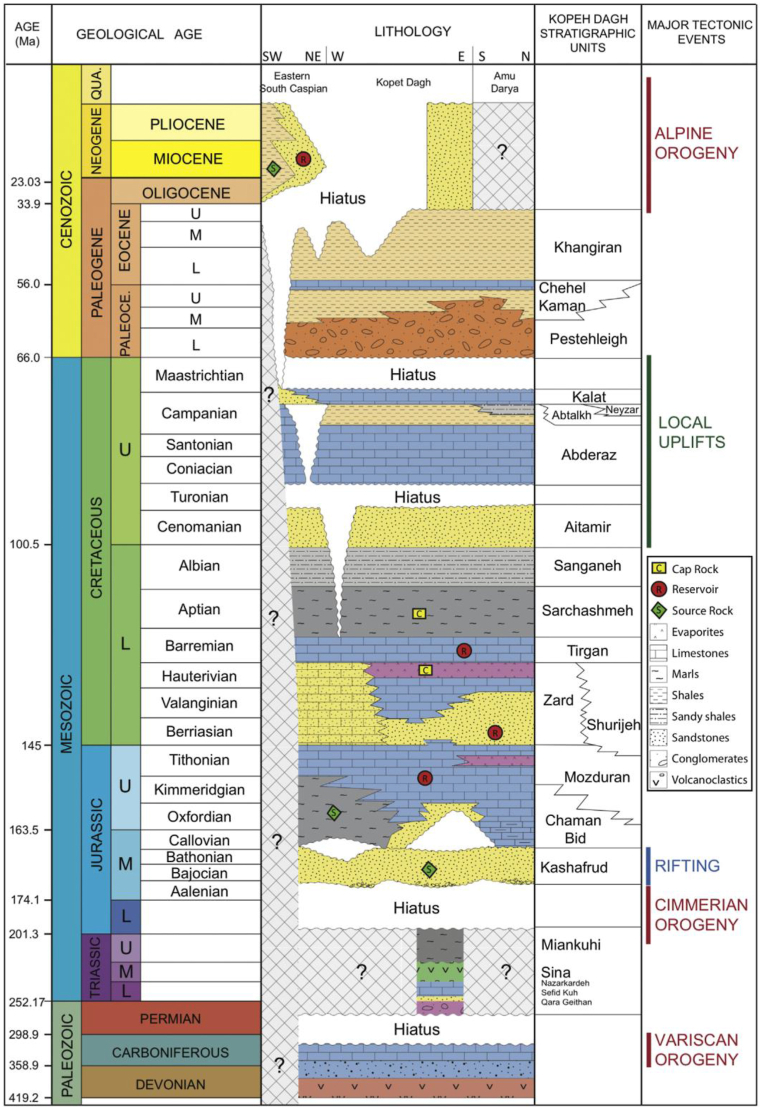
Stratigraphic chart of the Kopeh Dagh belt, Amu Darya and South Caspian Sea basins showing units, major unconformities, and correlation with tectonic events [38].

The complex folding, thrust faults, and structural development of the Kopeh Dagh Basin offer several opportunities for the deposit of hydrocarbons. Continued investigation is expected to result in additional findings, solidifying the basin's status as a globally renowned oil and gas location linked to the larger Amu Darya area [38,44,45]. The intricate arrangement of rock layers and the geological movements are linked between the Kopeh Dagh, Amu Darya, and South Caspian regions, as depicted in Fig. 4's stratigraphic chart.

## 3 Material and methods

### 3.1 Material

This study utilizes well logs and lost circulation data obtained from three wells (Wells A, B, and C) located in a gas field within the Kopeh Dagh basin. The dataset included a range of log data to evaluate various properties of the rock formation. These measurements included bulk density (RHOB, kg/m3) to determine the density of the rock, photoelectric factor (PEF, b/e) to evaluate the rock's resistance to photon penetration, neutron porosity (NPHI, v/v) to estimate the porosity of the formation based on its hydrogen content, shallow resistivity (LLS, ohm.m) and deep resistivity (LLD, ohm.m) to measure the electrical resistance near and far from the borehole, spectral gamma-ray (SGR, gAPI) to measure the natural radioactivity of the rocks, acoustic travel time (DT, us/m) to represent the interval transit time of sonic waves, computed gamma-ray (CGR, gAPI) derived from spectral measurements, caliper (CALI, mm) to measure the diameter of the borehole, and lost circulation (Mud Loss, bph) to quantify the amount of drilling mud lost from fractures or cavities.

The dataset contained 1662 data points that were acquired through wireline recording with water-based mud. Well A contributed 635 data points from a depth range of 2900.01–3759.09 m, Well B contributed 483 data points from a depth range of 2626.76–4004.91 m, and Well C contributed 544 data points from a depth range of 2675.07–3539.03 m. The dataset is statistically summarized in Table 2, which includes the total number of samples, mean, standard deviation, minimum, 25th percentile, median (50th percentile), 75th percentile, and maximum values.

**Table 2:** Statistical summary of the dataset.

Parameter	Count	Mean	Std	Min	25 %	50 %	75 %	Max
CALI (mm)	1662	283.99	51.39	203.43	220.42	316.35	320.21	401.33
CGR (gAPI)	1662	36.98	30.51	0.38	5.95	33.50	60.85	121.02
SGR (gAPI)	1662	43.24	32.05	2.92	9.68	41.60	67.59	127.62
DT (us/m)	1662	195.45	23.62	145.67	174.98	194.10	213.56	312.07
LLD (ohm.m)	1662	172.48	309.57	5.81	15.93	39.40	198.61	2915.98
LLS (ohm.m)	1662	196.93	386.39	5.49	17.87	47.85	159.03	1950.00
NPHI (v/v)	1662	0.07	0.05	−0.01	0.03	0.06	0.11	0.23
PEF(b/e)	1662	4.41	1.18	2.83	3.55	4.01	4.94	9.14
RHOB (kg/m3)	1662	2703.46	85.81	2093.64	2663.00	2723.68	2752.76	2971.99
Mud Loss (bph)	1662	49.02	71.88	0.00	2.00	25.00	50.00	200.00

**Fig. 5:**
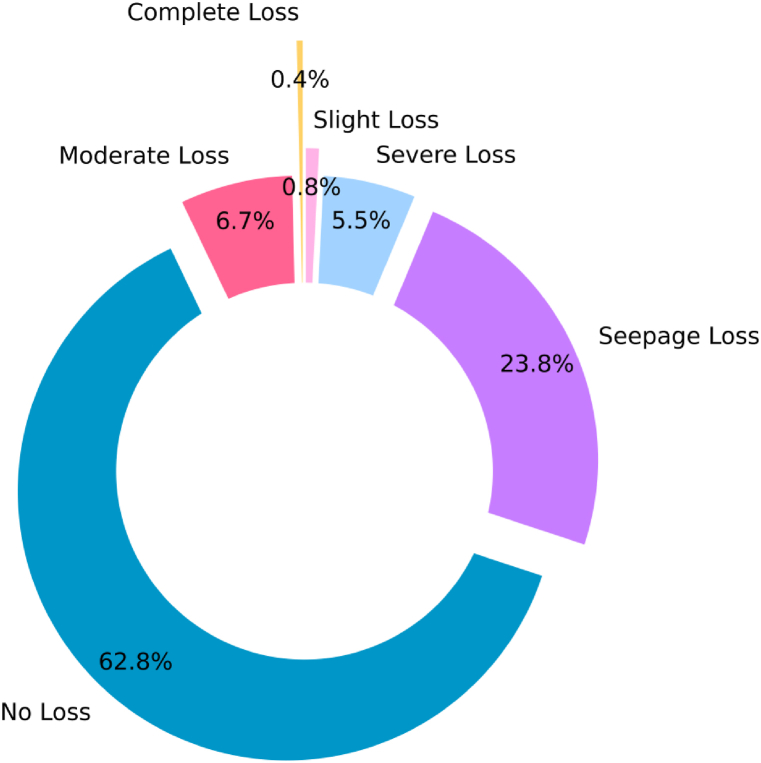
Distribution of various loss circulation categories.

Analysis of the data revealed six separate discrete categories defining the degree of drilling fluid circulation loss events. Complete Loss (6 samples), Moderate Loss (112 samples), Severe Loss (92 samples), Slight Loss (13 sample), Seepage Loss (395 sample), and No Loss (1044 sample) were the classification for these loss events. Fig. 5 shows how differently data records are distributed among these lost circulation categories. Out of the data, about 62.8 % fall into the No Loss group. Less than 2 % of the dataset is contained in the Complete Loss and Slight Loss categories taken together. The unequal distribution of samples emphasizes the need of applying sampling methods to consider the minority groups while building prediction models with this dataset.

**Fig. 6:**
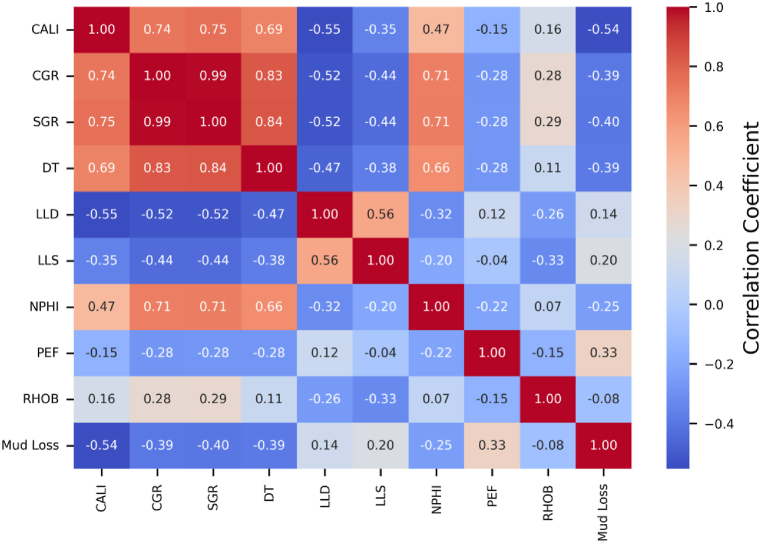
Cross-correlation matrix plot of 9 well logs and Mud Loss.

Fig. 6 displays the cross-correlation matrix between the nine well logs and the lost circulation rate (Mud Loss) investigated in this study. This matrix provides important new perspectives on the interactions between lost circulation and well-log measurements. As seen in the correlation matrix, Mud Loss shows specifically correlation coefficients between −0.54 and 0.33 with the several well-log parameters. The strong negative correlation of −0.54 between Mud Loss and DT suggests an inverse relationship whereby reduced sonic travel durations are linked to increased lost circulation. The positive correlation of 0.33 between Mud Loss and PEF implies that more important lost circulation is connected with greater photoelectric factors.

Additionally, LLD and LLS exhibit negative correlations of around −0.5 with CALI, CGR, and SGR. Deep and shallow resistivity decreases as the borehole diameter, gamma ray, and spectral gamma ray increase. On the other hand, above 0.7, the caliper, gamma ray, and spectral gamma ray are all positively correlated—that is, they rise and fall together. These relationships highlight the complex interactions among the several well logs that must be taken into account when modeling and forecasting lost circulation occurrences during drilling.

### 3.2 Data preparation

#### 3.2.1 Oversampling

The Adaptive Synthetic Sampling (ADASYN) algorithm is a key data preprocessing method used to fix class imbalance, which is a big problem when modeling the lost circulation in this study [46]. ADASYN is commonly employed in machine learning scenarios where there is a significant imbalance across classes. This solution works by dynamically creating artificial minority instances, addressing the imbalance issue by selectively increasing the number of underrepresented classes. ADASYN has been acknowledged for its efficacy in addressing imbalances that traditional oversampling methods may struggle with [47].

To implement ADASYN, the first step is to initialize the algorithm and define important parameters such as the sampling technique and the number of nearest neighbors for oversampling. Subsequently, it goes through a series of iterative processes linked to each class label. These iterations consist of dividing classes into training and temporary testing sets, and the combined temporary test sets allow for comprehensive evaluation. The main objective of ADASYN is to generate more samples for each class, resulting in a training dataset that is balanced. Following the process of oversampling, each class achieved a total of 622 samples, resulting in a substantial enhancement of the model's ability to generalize and perform well on this balanced dataset.

#### 3.2.2 Dataset standardization

This work has standardized features as a preprocessing step to scale them within a similar range so that not all input features have different scales and contribute equally to the predictive model. In addition, this helps the convergence to be fast. There were nine input variables: CALI, CGR, SGR, DT, LLD, LLS, NPHI, PEF, and RHOB, and Mud Loss was considered a target variable. Only the feature set was subjected to standardization, while the target variable, Mud Loss, was allowed to remain on its original scale. The standardization will be done using the method of a z-score, wherein a particular feature is transformed into a mean of 0 and a standard deviation of 1. This was very important for algorithms reliant on gradient-based optimization or distance metrics since it would introduce bias if there were large differences in the feature scales. All the features were standardized according to the formula in Eq. (1).

(1) $$X_{\text{Standardized}}=\frac{X-\mu }{\sigma }$$

Where *X* represents the original value of the feature, *μ* denotes the mean of the feature across all samples, and σ is the standard deviation of the feature.

#### 3.2.3 Feature selection

Recursive Feature Elimination (RFE) was used in conjunction with grid search to systematically pick features during the development of machine learning classifiers. Recursive Feature Elimination (RFE) is a powerful method that iteratively removes the least significant features, leaving only the most useful attributes intact [48,49]. In order to carry out Recursive Feature Elimination (RFE), pipelines were created for each classifier, consisting of the RFE module and the classifier itself. This systematic approach allowed for the simultaneous optimization of both feature selection and hyperparameters.

The pipelines methodically assessed potential feature counts using grid search. By utilizing cross-validation and evaluating the macro F1-score, we guaranteed that the selected features could be effectively applied to different data sets, maintaining a balance between precision and recall. The grid search identified the most suitable number of features and adjusted the pipeline accordingly. Subsequently, the improved pipeline was employed to make predictions on the test dataset. The procedure of selecting features increased the model's generalization by prioritizing the most informative ones.

Table 3 illustrates the effectiveness of various classifiers, showcasing the results of feature selection. The objective was to identify the most effective subset of features for a precise prediction model while considering the trade-off between reducing the number of dimensions and retaining relevant information. The Extra Trees model had the highest performance, with a macro F1-score of 0.9, utilizing only seven features. On the other hand, Random Forest and SVM classifiers utilized a greater number of features but achieved lower F1 scores. The results demonstrate Extra Trees' efficacy in selecting a subset of highly informative features, thereby improving prediction accuracy while handling the issue of high dimensionality.

**Table 3:** Comparison of feature selection and model performance across classifiers.

Classifier	Best Features	Selected Features	Macro F1-Score
Random Forest	9	CALI, CGR, SGR, DT, LLD, LLS, NPHI, PEF, RHOB	0.79
Extra Trees	7	CALI, CGR, SGR, DT, LLD, LLS, PEF	0.90
XGBoost	9	CALI, CGR, SGR, DT, LLD, LLS, NPHI, PEF, RHOB	0.79
Decision Tree	8	CALI, CGR, SGR, DT, LLD, LLS, PEF, RHOB	0.78
SVM	9	CALI, CGR, SGR, DT, LLD, LLS, NPHI, PEF, RHOB	0.47
k-NN	9	CALI, CGR, SGR, DT, LLD, LLS, NPHI, PEF, RHOB	0.71

### 3.3 Methods

The approach for forecasting the severity of lost circulation utilizes many machine-learning algorithms to produce reliable forecasts. The prediction skills of Random Forest, Extra Trees, XGBoost, Decision Trees, Support Vector Machine (SVM), and k-Nearest Neighbors (k-NN) are specifically utilized. Each of these fundamental learners provides distinct perspectives on the situation.Initially, the base learners undergo training using a dataset consisting of well logs and lost circulation data. Following that, the trained algorithms generate autonomous forecasts for the intensity of lost circulation. These individual predictions encompass a wide range of viewpoints on the issue.Subsequently, the base learner's predictions are combined using the hard-voting ensemble technique. Hard Voting is a technique that takes the predicted classes from many base models and determines the final prediction based on the class that receives the most votes. This allows the combined intelligence derived from a wide range of base models to be utilized.The primary benefit of this strategy is its ability to combine the advantages of many modeling techniques in order to provide precise and reliable forecasts. By utilizing tree-based algorithms, Support Vector Machines (SVM), and k-Nearest Neighbors (k-NN) models within a structured framework, it is possible to accurately consider intricate relationships within the data. The variety included in the ensemble also enhances the ability to generalize and safeguards against overfitting.

#### 3.3.1 Random forest

Random Forest is an adaptable ensemble learning technique that combines the knowledge of several decision trees [50]. Every tree in the ensemble is trained on a random sample of data points and attributes, which introduces variability. This variability reduces the correlation between trees and improves generalization while also limiting overfitting [51]. The ultimate forecast of the Random Forest model is obtained by combining the results of these independent trees, usually through averaging. This process creates a strong learner from a collection of weaker learners. To enhance performance, one can fine-tune additional hyperparameters, such as the number of trees and subset sizes. Random Forest is very versatile, scalable, and robust, making it an excellent option for classification and regression problems in several fields. This has led to its widespread use and acceptance [52,53].

#### 3.3.2 Extra Trees

Originally known as Extremely Randomized Trees, Extra Trees is a sibling of Random Forest that adds yet another degree of unpredictability [54]. Extra Trees randomly choose feature splits instead of looking for the best split, unlike regular decision trees. This additional source of randomness, mainly in relation to noisy or high-dimensional data, acts as a defensive mechanism against overfitting, thereby improving predictive accuracy [55]. Extra Trees' inclination for randomness and ensemble learning ability produce a consistent model capable of managing difficult datasets [54].

#### 3.3.3 XGBoost

Incorporating a spectrum of techniques, including regularization, tree pruning, and a customized loss function, XGBoost—also known as Extreme Gradient Boosting—stands out as an efficient and scalable gradient boosting method to finely tune model performance [56,57]. Celebrated for its extraordinary speed and accuracy, XGBoost has become a strong rival in data science contests and a preferred choice in academic research and industry applications ranging over the machine learning spectrum. XGBoost has been enabled to shine across an array of regression, classification, and ranking issues involving large-scale and complex datasets by its computational efficiency, innovative prediction capabilities, and adaptability via hyperparameter adjustment [58].

#### 3.3.4 Decision tree

A basic machine learning method, the Decision Tree offers a simple yet effective method for addressing classification and regression problems [59]. It arranges data into a hierarchical network of nodes, each of which reflects a choice based on a feature, therefore producing a final prediction. Transparency provided by decision trees helps to clearly grasp feature significance. To prevent overfitting and excel in difficult tasks, they do, however, need careful pruning and usually benefit from the support of ensemble approaches [60]. Particularly coupled with ensemble techniques, its simple structure, interpretability, and ability to capture nonlinear interactions have confirmed decision trees as a flexible supervised learning method [61].

#### 3.3.5 Support vector machine

The Support Vector Machine (SVM) is a powerful supervised learning technique used for classification and regression tasks [62]. It creates hyperplanes in spaces with several dimensions that are excellent for separating classes or fitting data trends to the maximum extent [63]. The support vector machine (SVM) is highly effective at representing complex nonlinear decision boundaries. It achieves this by utilizing kernel methods to transform inputs into feature spaces of higher dimensions [64]. The reason for its resistance to overfitting is due to the maximal margin characteristic. Support Vector Machines (SVM) have excellent performance on datasets of small to medium sizes and are highly effective even when dealing with feature sets that have a low density of data points. The complexity of the model is controlled by soft margin regularization, making the Support Vector Machine (SVM) versatile and adaptive [62,64].

#### 3.3.6 K-nearest neighbors

The k-Nearest Neighbors (k-NN) represents a straightforward yet effective non-parametric technique for handling classification and regression tasks [65,66]. Based on their closeness to k's nearest neighbors in the training set, it forecasts fresh data points. By considering local data neighbors, the k-NN can represent intricate nonlinear decision boundaries without assuming any form of shape of decision boundary [67]. It offers interpretability via nearest neighbors and fits complex trends in data. Model smoothness and complexity are under control by the hyperparameter k. All things considered, k-NN is a flexible method good in using local data structures [66,67].

#### 3.3.7 Voting

Voting is a potent ensemble technique that amalgamates predictions from multiple base models to generate robust final predictions [68]. Two primary variants of voting are Hard Voting and Soft Voting. In Hard Voting, the predicted class labels from base models are aggregated, and the class with the majority of votes is selected as the ensemble prediction. This simple majority rule leverages the strengths of different models. Soft Voting extends this concept by weighting base model predictions according to their confidence scores [69]. Class probabilities are averaged, and the class with the highest average probability is predicted, accounting for model uncertainty. While Soft Voting typically outperforms Hard Voting, it is computationally more intensive [70]. Voting amalgamates diverse model perspectives, enhancing ensembles' stability, accuracy, and generalization. The voting framework provides a flexible means of harnessing collective knowledge from a group of base learners [68,69]. Table 4 supplies a comprehensive overview of the hyperparameters utilized in the models.

**Table 4:** Overview of hyperparameters employed in base models.

Model	Hyperparameters
Random Forest	n_estimators = 100, max_depth = 10, max_features = sqrt, criterion = gini, random_state = 42
Extra Trees	n_estimators = 100, max_depth = 10, max_features = sqrt, criterion = gini, random_state = 42
XGBoost	learning_rate = 0.1, max_depth = 3, n_estimators = 100, random_state = 42
Decision Tree	max_depth = 5, criterion = gini, random_state = 42
SVM	kernel = rbf, C = 1.0, gamma = scale, random_state = 42
k-NN	n_neighbors = 5, weights = uniform, p = 2
Voting	estimators = individual_classifiers, voting = hard

## 4 Results

This section delivers the results obtained from the machine learning models employed in this study. Multiple evaluation metrics and visualizations are leveraged to assess the performance of the models. The aim is to scrutinize the comparative advantages and limitations of the differing modeling methodologies to ascertain which approach generates the most precise predictions of lost circulation intensity.

### 4.1 Data partitioning

The complete dataset was partitioned into two discrete subsets to procure a reliable assessment of the model's capabilities. The first subset, comprising 70 % of the data, was utilized to train the model. The second subset, encompassing the remaining 30 % of the data, functioned as an independent test set. This separation into distinct training and testing sets was performed randomly. Employing an independent test set assists in mitigating the possibility of overfitting, as the test data furnishes an unbiased estimate of the trained model's capacity to generalize to novel, unseen data. This approach of segregating the data into separate training and test sets facilitates a robust evaluation of out-of-sample predictive performance.

### 4.2 Model evaluation metrics

Using specified measures, the classification model's performance is assessed. These metrics are crucial for evaluating the model's lost circulation intensity prediction. The model's accuracy is the ratio of accurately predicted instances to the total number of instances. Precision, or positive predictive value, is the percentage of model-generated positive predictions that are correct. It shows the model's positive case classification accuracy. The ratio of true positives to total positive instances is recall, also known as sensitivity or true positive rate. It measures the model's ability to find all relevant instances. The harmonic mean of precision and recall, the F1-Score, balances these two key parameters, making it effective for imbalanced datasets. Using all four confusion matrix categories, the Matthews Correlation Coefficient (MCC) quantifies the link between observed and predicted classifications. It is a reliable classification quality metric in binary and multi-label situations [71]. Cohen's Kappa evaluates the agreement between actual and anticipated labels, taking into account chance [72]. Hamming Loss, the fraction of wrongly assigned labels across samples, measures multi-label classification prediction quality [73]. However, the Jaccard Index compares predicted and actual label sets, with higher values suggesting better multi-label classification [74]. The mathematical expressions for each metric are presented in Table 5.

**Table 5:** Equations for evaluation metrics in classification model performance assessment.

Metric	Equation	Term
Accuracy	$\frac{Number\;of\;Correct\;Predictions}{Total\;Number\;of\;Predictions}$	–
Precision	$\frac{True\;Positives}{True\;Positives+False\;Positives}$	True Positives: Correctly predicted positives False Positives: Incorrectly predicted positives
Recall	$\frac{True\;Positives}{True\;Positives+False\;Negatives}$	
F1-Score	$\frac{2\cdot Precision\cdot Recall}{Precision+Recall}$	–
MCC	$\frac{TP\cdot TN-FP\cdot FN}{\sqrt{\left(TP+FP\right)\left(TP+FN\right)\left(TN+FP\right)\left(TN+FN\right)}}$	TP, TN, FP, FN: Count of true positives, true negatives, false positives, and false negatives
Cohen's Kappa	$\frac{Observed\;Agreement-Expected\;Agreement}{1-Expected\;Agreement}$	Observed Agreement: Actual agreement between predictions Expected Agreement: Agreement expected by chance
Hamming Loss	$\frac{1}{N}\sum_{i=1}^{N}\frac{1}{M}\sum_{j=1}^{M}\delta \left(y_{ij}\neq {\hat{y}}_{ij}\right)$	N: Total instances M: Total labels $\delta \left(y_{ij}\neq {\hat{y}}_{ij}\right)$: Measures label differences
Jaccard Index	$\frac{Size\;of\;Intersection\;between\;Actual\;and\;Predicted\;Sets}{Size\;of\;Union\;between\;Actual\;and\;Predicted\;Sets}$	Intersection: Common labels between actual and predicted sets Union: All labels in actual and predicted sets

### 4.3 Performance of base models

**Fig. 7:**
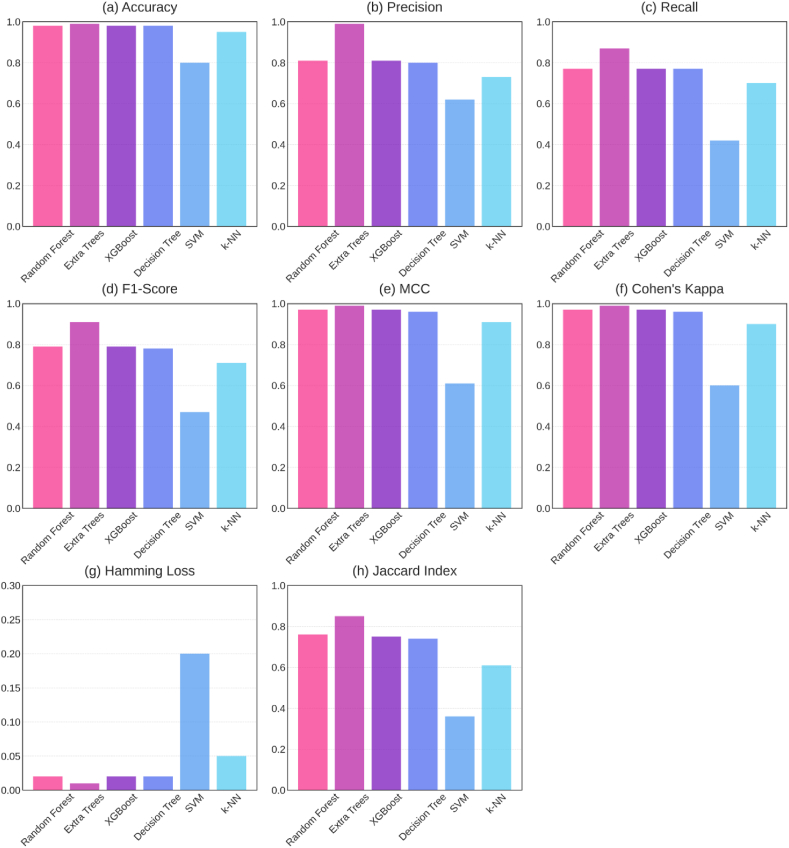
A visual overview of model performance metrics (a) Accuracy, (b) Precision, (c) Recall, (d) F1-Score, (e) MCC, (f) Cohen's Kappa, (g) Hamming Loss, (h) Jaccard Index.

This section evaluates the performance of lost circulation intensity prediction by six base classifiers on the dataset, employing the mentioned metrics. Specifically, the classifiers under scrutiny encompass Random Forest, Extra Trees, XGBoost, Decision Tree, Support Vector Machine (SVM), and k-Nearest Neighbors (k-NN). The outcomes of this performance evaluation for various machine learning classifiers applied to the dataset are detailed in Table 6 and visually represented in Fig. 7.

**Table 6:** Performance of base models.

Classifier	Accuracy	Precision	Recall	F1-Score	MCC	Cohen's Kappa	Hamming Loss	Jaccard Index
Random Forest	0.98	0.81	0.77	0.79	0.97	0.97	0.02	0.76
Extra Trees	0.99	0.98	0.86	0.90	0.98	0.98	0.01	0.83
XGBoost	0.98	0.81	0.77	0.79	0.97	0.97	0.02	0.75
Decision Tree	0.98	0.80	0.77	0.78	0.96	0.96	0.02	0.74
SVM	0.80	0.62	0.42	0.47	0.61	0.60	0.20	0.36
k-NN	0.95	0.73	0.70	0.71	0.91	0.90	0.05	0.61

**Fig. 8:**
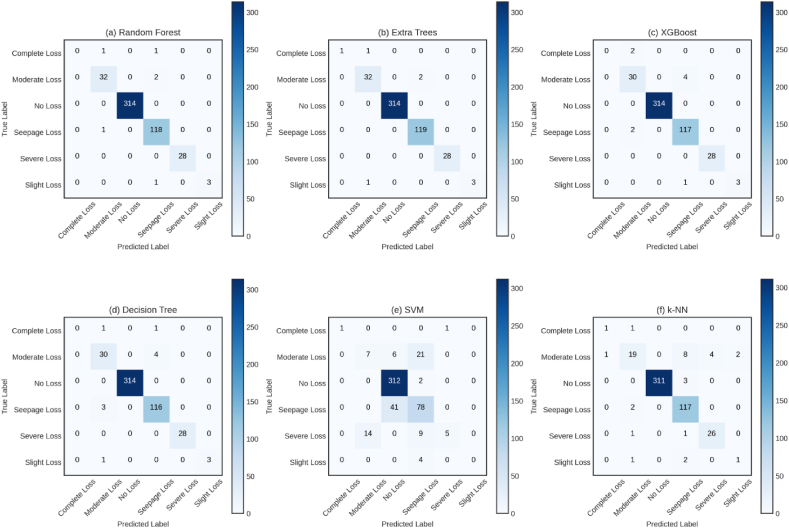
Confusion matrices for six base machine learning models in loss classification (a) Random Forest, (b) Extra Trees, (c) XGBoost, (d) Decision Tree, (e) SVM, and (f) k-NN.

With regard to general performance, ensemble techniques such Random Forest and Extra Trees have shown the most positive outcomes for estimating lost circulation intensity. With an accuracy of 0.99, precision of 0.98, recall of 0.86, F1-score of 0.90, Matthews Correlation Coefficient (MCC) of 0.98, Cohen's Kappa value of 0.98, and Jaccard Index of 0.83 Extra Trees had especially outstanding performance. Moreover, it reached the lowest Hamming Loss value of 0.01. Random Forest produced likewise commendable outcomes with metrics somewhat in line with those of Extra Trees. With an accuracy of 0.98 compared to XGBoost's 0.98 for lost circulation intensity prediction, Decision Tree did somewhat poorly among the individual tree-based techniques. Furthermore, whilst XGBoost showed a precision of 0.81, Decision Tree produced a precision of 0.80. Recall saw both Decision Tree and XGBoost record scores of 0.77. XGBoost displayed, nevertheless, a somewhat better F1-score (0.79 vs. 0.78), MCC (0.97 vs. 0.96), Cohen's Kappa (0.97 vs. 0.96), and Jaccard Index (0.75 vs. 0.74). With relation to Hamming Loss, both approaches registered a value of 0.02. On the other hand, the SVM and k-NN classifiers showed less good performance than the tree-based approaches in forecasting loss circulation intensity. Particularly, SVM showed a less-than-ideal F1-score of 0.47 and the recall, scoring just 0.42, showed a clear lacking. By k-NN, on the other hand, a rather better recall of 0.70 was obtained; however, it lagged behind the best-performing approaches. In terms of performance across all assessment criteria used to forecast lost circulation intensity in this dataset, the ensemble approaches—more especially, Extra Trees and Random Forest—clearly ranked. While SVM and k-NN clearly showed poorer performance, the tree-based algorithms exceeded other techniques. Six base machine learning models are evaluated holistically in Fig. 8 using confusion matrices. Evaluating model performance across six different classes—Complete Loss, Moderate Loss, No Loss, Seepage Loss, Severe Loss, and Slight Loss—these matrices are quite useful instruments.

The capability of accurately classifying instances of “Moderate Loss” and “No Loss” is demonstrated by the Random Forest Confusion Matrix. However, it is occasionally found to stumble when dealing with “Complete Loss” and “Seepage Loss.” It sometimes experiences challenges distinguishing between “Slight Loss” and “Moderate Loss.” Similarly, commendable performance in accurately classifying “Moderate Loss” and “No Loss” instances, with fewer misclassifications of “Complete Loss” and “Seepage Loss” compared to Random Forest, is demonstrated by the Extra Trees Confusion Matrix. Nevertheless, it is still observed to distinguish between “Slight Loss” and “Moderate Loss."

Moving on to the XGBoost Confusion Matrix, an enhanced ability to classify “Complete Loss” is showcased compared to the previous models. However, it continues to grapple with distinguishing between “Slight Loss” and “Moderate Loss” and occasionally falters in identifying instances of “Seepage Loss.” The performance of the Decision Tree Confusion Matrix parallels that of XGBoost and Random Forest, excelling in accurately classifying “Moderate Loss” and “No Loss.” Nevertheless, it faces difficulties in distinguishing between “Slight Loss” and “Moderate Loss” and sporadically misclassifies “Complete Loss” and “Seepage Loss."

In contrast, the SVM Confusion Matrix displays a different classification pattern compared to the ensemble methods. It effectively categorizes “Moderate Loss” and “No Loss” but grapples with “Seepage Loss.” Additionally, it encounters challenges in distinguishing between “Complete Loss” and “Slight Loss” and occasionally misclassifies “Severe Loss.” Lastly, a strong proficiency in classifying “Moderate Loss” instances is demonstrated by the k-NN Confusion Matrix, but it faces hurdles with “Complete Loss” and “Seepage Loss.” Like other models, it struggles to differentiate between “Slight Loss” and “Moderate Loss."

**Fig. 9:**
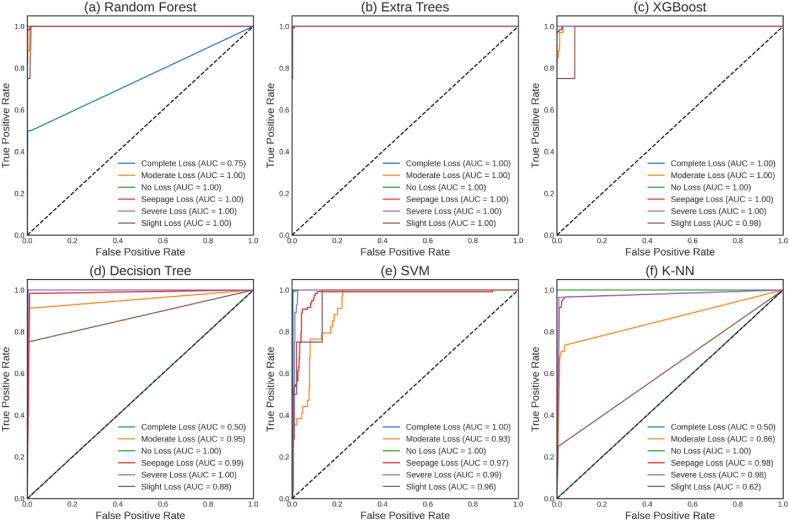
ROC Curve for various base models: (a) Random Forest, (b) Extra Trees, (c) XGBoost, (d) Decision Tree, (e) SVM, and (f) k-NN.

Fig. 9 illustrates the evaluation of Receiver Operating Characteristic (ROC) curves for six base machine learning models, offering a visual representation of the trade-off between true positive rate (sensitivity) and false positive rate (1-specificity) in assessing classification model performance. The models were rigorously assessed across six lost circulation intensity categories, spanning from “Complete Loss” to “Slight Loss,” enabling a comprehensive examination of their performance under different levels of lost circulation intensity. To interpret the results presented in Fig. 9, the focus is placed on the area under the ROC curve (AUC) as a critical performance metric. The AUC quantifies the overall discriminative power of a model, with superior predictive performance indicated by a higher AUC. Perfect classification is represented by a model with an AUC of 1.0, while an AUC of 0.5 corresponds to random guessing.

An analysis of the ROC curve and corresponding AUC values for each model yields significant and novel insights. The Extra Trees and XGBoost models got a perfect AUC of 1.0 for Complete Loss prediction, demonstrating their flawless discriminating abilities. The Random Forest and SVM models demonstrated exceptional performance, with AUC values of 0.75 and 1.0, respectively. Regarding Moderate Loss, each model has exhibited outstanding performance. The AUC values for the k-NN model exhibited a range of 0.86, whereas the Extra Trees, XGBoost, and SVM models had a range of 1.0. All models accurately predicted the absence of lost circulation, as indicated by a perfect AUC value of 1.0, demonstrating complete accuracy in anticipation. The Extra Trees, XGBoost, and SVM models had exceptional predictive abilities for Seepage Loss, as evidenced by their AUC values of 1.0, 1.0, and 0.97, respectively. The Extra Trees and XGBoost models demonstrated excellent performance in handling Severe Loss, as indicated by their AUC of 1.0. The SVM model achieved an impressive AUC of 0.99. The Extra Trees model outperformed the other models in predicting Slight Loss, achieving an AUC of 0.96. While XGBoost, SVM, and Random Forest yielded satisfactory results, the k-NN model exhibited subpar performance in this domain.

The ROC curve analysis highlights the consistent and strong predictive capability of the Extra Trees and XGBoost models across various levels of lost circulation intensity. In addition to SVM and Random Forest, these models, which have AUC values close to or equal to 1.0, demonstrate significant potential as valuable tools for evaluating the intensity of lost circulation in drilling operations.

### 4.4 Hard Voting performance

In this section, the performance of the Hard Voting classifier is rigorously assessed utilizing a suite of fundamental metrics encompassing accuracy, precision, recall, F1-score, Matthews Correlation Coefficient (MCC), Cohen's Kappa, Hamming Loss, and Jaccard Index. This meticulous examination is conducted on a dataset comprising six base classifiers (Table 7).

**Table 7:** Performance of hard voting.

Classifier	Accuracy	Precision	Recall	F1-Score	MCC	Cohen's Kappa	Hamming Loss	Jaccard Index
Hard Voting	0.99	0.99	0.87	0.91	0.99	0.99	0.01	0.84

With a score of 0.99, the Hard Voting's performance stands out for being exceptionally accurate. This demonstrates the group's exceptional skill in correctly classifying 99 % of cases. Moreover, the precision score of 0.99 of the ensemble validates its ability to reduce false positives, providing a high level of trust in its predictions. While the memory score of 0.87 indicates an excellent recollection of positive examples, there is still room for improvement in this area. The ensemble's F1-score of 0.91 shows that it has achieved an excellent balance between recall and precision, demonstrating its proficiency in both accurate positive classifications and the reduction of false positives.

The remarkable agreement between the Hard Voting results and real labels is amplified by the MCC score of 0.99 and Cohen's Kappa score of 0.99. Notably, the Hamming Loss is a very low value of 0.01; this indicates that there are very few incorrect predictions in any class. Finally, the Jaccard Index score of 0.84 emphasizes how well the Hard Voting predictions match the real class labels, particularly considering the complexity of the multi-class situation.

**Fig. 10:**
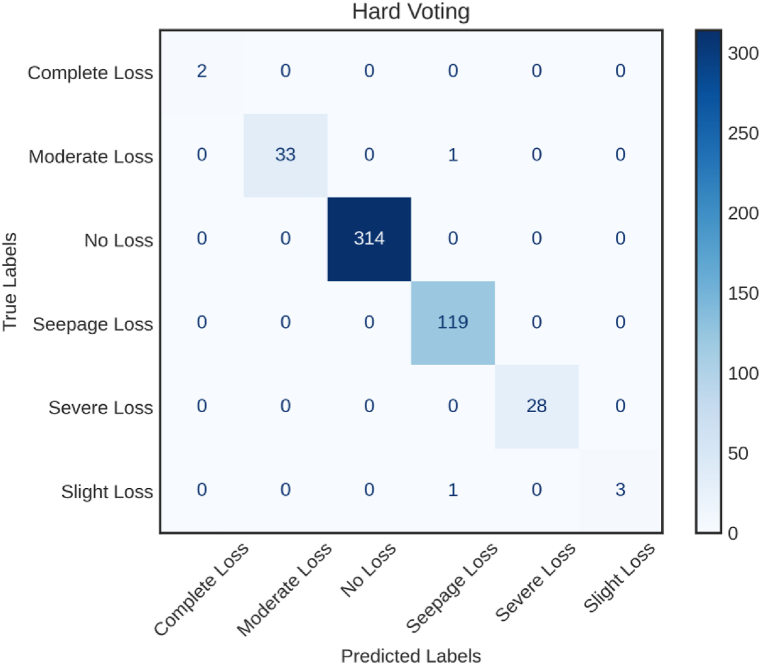
Confusion matrices for Hard Voting.

As demonstrated in Fig. 10, the confusion matrix becomes a crucial tool for assessing the per-class classification performance of the Hard Voting. It goes beyond general accuracy by providing a detailed comprehension of the model's advantages and disadvantages in various categories. Specifically, the ensemble excels at reducing classification mistakes in the “Complete Loss” and “Moderate Loss” classes, which is important in situations where misclassification could result in expensive and perhaps dangerous consequences. The Hard Voting's ability to recognize cases in the “No Loss” and “Seepage Loss” categories highlights how effective it is. However, there is room for refinement in classifying “Slight Loss” instances, as indicated by non-zero values in the corresponding cell.

**Fig. 11:**
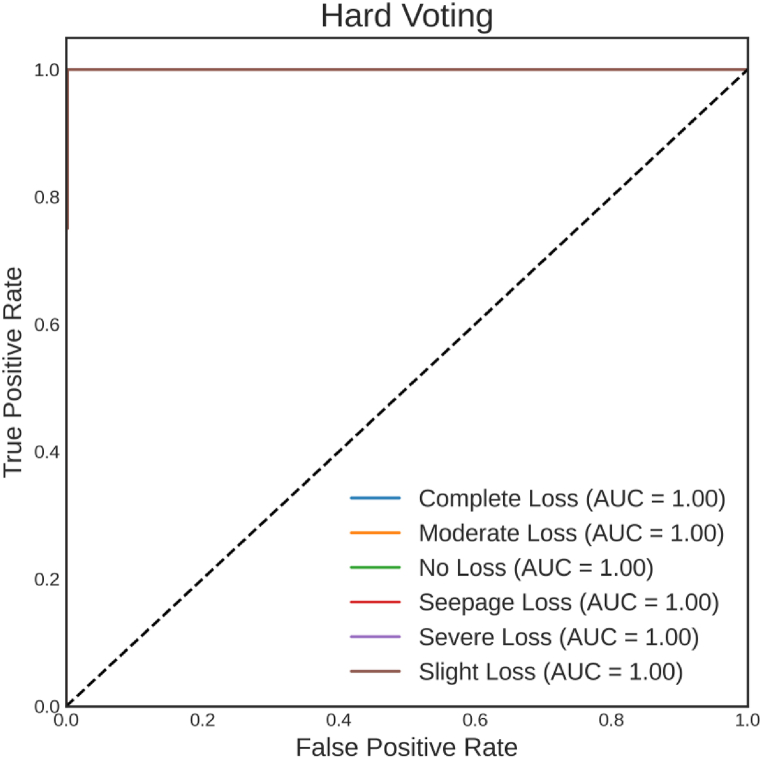
ROC curve for Hard Voting.

Fig. 11 illustrates the ROC curve of the Hard Voting. This analysis offers an extensive perspective on the ensemble's discriminatory capabilities. Its AUC of 1 for all classes underscores the Hard Voting's extraordinary ability to distinguish between categories. This remarkable result signifies that Hard Voting's predictions yield high true positive rates and low false positive rates across all classes. This equates to consistently reliable decisions and heightened effectiveness in correctly identifying each class. This perfect separation between classes substantiates the Hard Voting's robustness and underscores its suitability for applications where precision in classification is paramount. The ROC curve unequivocally highlights the impressive performance of Hard Voting in this classification task.

In conclusion, the outstanding performance of the Hard Voting, amalgamating predictions from six diverse base classifiers, is exhibited across an array of evaluation metrics. Its excellence in accuracy, precision, MCC, Cohen's Kappa, and Hamming Loss establishes it as a promising choice for the classification task. While there is room for improvement in recall for specific classes, such as “Complete Loss,” the overall results affirm that Hard Voting is a robust and dependable classifier for this particular problem.

## 5 Discussion

The comprehensive empirical evaluation conducted in this study offers valuable insights into the selection of optimal machine-learning techniques for building resilient and transferable systems capable of accurately predicting the severity of lost circulation in the context of intricate drilling operations. Furthermore, this section deals more elaborately with the reasoning behind the findings, bringing out considerable ramifications and the importance of the results obtained, especially those related to ensemble-based methods. Extra Trees and Hard Voting are the most prominent competitors in this regard. However, the foundation of this question is indeed Extra Trees, which has surely turned out to be very proficient over a wide set of performance measures. Its architecture of building multiple decision trees out of subsets of the data and features allows it to achieve high variance reduction with improved generalization. Success in the model could be realized by capturing the complex interaction among the features inherent in the prediction of a multi-class problem like the intensity of lost circulation.

What is more, robustness against overfitting enhances this success. Indeed, this reflects the overall efficacy of the model, given the performance metrics: accuracy, precision, recall, F1-score, Matthews Correlation Coefficient, Cohen's Kappa, and Jaccard Index. The notable Hamming Loss of 0.01 shows that it is pretty accurate in categorizing even the most stubborn intensity levels in complete and seepage loss. Further, this capability underlines the critical role it could play in practical applications where correct predictions are vital.

Besides, the Hard Voting ensemble, which even further combines the strengths of six different base classifiers, has also achieved performance in every important criterion equal to or slightly above that of the Extra Trees model. Strong evidence for the robust nature of the performed predictions by the ensemble is underlined by the superb scores of 0.99 regarding various metrics that highlight the performance. This is attributed to the diversity in nature of the base classifiers since the ensemble can make amends for weaknesses and build on the strengths. Its exceptional AUC value of 1.0 for all classes of intensities further cements its reliability. The confusion matrix provides valuable insight into how improvements in specific areas are made, especially for the more difficult categories like Slight Loss. These observations testify to the ensemble's flexibility and the fact that further improvements might be achieved by applying more sophisticated base estimators and advanced sampling methods. Whereas, against more basic methods like Support Vector Machines and k-nearest Neighbors, it does much worse comparatively regarding metrics. This discrepancy underlines the shortage of these classical algorithms when solving the intrinsic, nonlinear relationships accompanying the prediction of lost circulation intensity. Their poor memory and F1 scores reflect their inability to capture the intricate patterns within the data, further confirming their inadequacy for this task. While classic tree-based algorithms, such as a single Decision Tree, have quite decent capabilities, they are noticeably outperformed by more modern algorithms, including XGBoost, boosting performance with gradient boost techniques. The advanced methods of the ensemble substantially outrank both Extra Trees and Hard Voting.

Receiver operating characteristic analysis confirms the goodness of Extra Trees and Hard Voting. Their exceptional AUC values ascertain the presence of great discrimination for all classes of intensity, even for the most complicated ones, underlining their capability to predict in shades. Ensemble methods are good for correct predictions and have a critical distinction between various important intensities for making lost circulation predictions.

These extensive results undoubtedly confirm the efficiency of ensemble learning methods, especially Extra Trees and Hard Voting techniques, as main approaches for lost circulation intensity predictions. Their excellent performance is reflected in several of the assessment indicators used, making them leading solutions to enable automated and data-driven analysis in drilling engineering. The reason the ensemble models could achieve such good performance is essential because of their inherent ability to combine multiple perspectives over the data, hence improving predictive accuracy and providing a wider view of lost circulation events.

These findings represent the contribution of this strict empirical study, setting new standards in the field and providing sound evidence that advanced ensemble learning methods, like Extra Trees and Hard Voting, are superior in creating precise, dependable, and widely applicable systems for predicting lost circulation intensity. Ensemble methods herein act as enablers of sophisticated AI solutions that will enable complex characterization tasks, open up many optimization opportunities, and lead to data-driven decisions in high-risk drilling operations. Their exceptional qualities are elicited through several evaluation methods, underlining their suitability to handle large, diversified datasets and their potential for implementation in real-world systems upon thorough domain adaptation. The findings reported in this research fundamentally change how lost circulation is managed and lay a sound foundation for innovative deep ensemble structures research in the future, opening enormous avenues for advancement in drilling engineering.

## 6 Conclusions

This study has indicated that well logs could present a feasible method for accurately predicting drilling operations regarding the intensity of lost circulation with the help of machine learning models. Among the surveyed techniques, the most robust results in key metrics were obtained using ensemble methods: Random Forest, Extra Trees, and Hard Voting. Then, the ensemble models used the cumulative prediction capabilities of their base estimators to cope with the complex nature of this multivariate prediction problem.

These ensemble methods had very promising potential for accurate characterization of lost circulation intensity in several classes and outperformed all individual techniques, XGBoost, Decision Tree, Support Vector Machine, and k-Nearest Neighbors. The application of ensemble methods will thus provide predictive intelligence for drillers to optimize operations and reduce risks associated with lost circulation.

This research effectively demonstrated the practicality of well logs and machine learning techniques in lost circulation prediction. This groundbreaking application considers improving lost circulation prediction in drilling engineering. The research has filled an important gap in the literature, as this is the first study that applies machine learning for lost circulation intensity prediction based on well logs. In that way, the research has provided a useful tool for the enhancement of drilling efficiency. Further work in this direction can lead to an improvement in operational safety, cost-effectiveness, and overall performance in drilling operations.

## Nomenclatures

This paper does not use any specialized nomenclatures that require definition.

**Table:** Acronyms

ADASYN	Adaptive Synthetic Sampling
ANN	Artificial Neural Network
AUC	Area Under the Curve
CALI	Caliper
CGR	Computed Gamma Ray
DT	Sonic Transit Time
FN	False negatives
FP	False Positives
k-NN	k-Nearest Neighbors
LLD	Deep Laterolog Resistivity
LLS	Shallow Laterolog Resistivity
MCC	Matthews Correlation Coefficient
NPHI	Neutron Porosity
PEF	Photoelectric Absorption Factor
RFE	Recursive Feature Elimination
RHOB	Bulk Density
ROC	Receiver Operating Characteristic
SGR	Spectral Gamma Ray
SVM	Support Vector Machine
TN	Count of true negatives
TP	Count of true positives
XGBoost	Extreme Gradient Boosting

## Data availability

The authors do not have permission to share the data.

## Statements and declarations

The authors declare that they have no known competing financial interests or personal relationships that could have appeared to influence the work reported in this paper.

## Funding

The authors did not receive support from any organization for the submitted work.

## Declaration of competing interest

The authors declare that they have no known competing financial interests or personal relationships that could have appeared to influence the work reported in this paper.
